# *Bordetella pertussis* outer membrane vesicle vaccine confers equal efficacy in mice with milder inflammatory responses compared to a whole-cell vaccine

**DOI:** 10.1038/srep38240

**Published:** 2016-12-01

**Authors:** René H. M. Raeven, Jolanda Brummelman, Jeroen L. A. Pennings, Larissa van der Maas, Wichard Tilstra, Kina Helm, Elly van Riet, Wim Jiskoot, Cécile A. C. M. van Els, Wanda G. H. Han, Gideon F. A. Kersten, Bernard Metz

**Affiliations:** 1 Institute for Translational Vaccinology (Intravacc), Bilthoven, The Netherlands; 2 Division of Drug Delivery Technology, Leiden Academic Centre for Drug Research, Leiden, The Netherlands; 3 Centre for Infectious Disease Control, National Institute for Public Health and the Environment (RIVM), Bilthoven, The Netherlands; 4 Centre for Health Protection (GZB), National Institute for Public Health and the Environment (RIVM), Bilthoven, The Netherlands

## Abstract

The demand for improved pertussis vaccines is urgent due to the resurgence of whooping cough. A deeper understanding of the mode of action of pertussis vaccines is required to achieve this improvement. The vaccine-induced effects of a candidate outer membrane vesicle vaccine (omvPV) and a classical protective but reactogenic whole cell vaccine (wPV) were comprehensively compared in mice. The comparison revealed essential qualitative and quantitative differences with respect to immunogenicity and adverse effects for these vaccines. Both vaccines stimulated a mixed systemic Th1/Th2/Th17 response. Remarkably, omvPV evoked higher IgG levels, lower systemic pro-inflammatory cytokine responses and enhanced splenic gene expression than wPV. The omvPV-induced transcriptome revealed gene signatures of the IFN-signaling pathway, anti-inflammatory signatures that attenuate LPS responses, anti-inflammatory metabolic signatures, and IgG responses. Upon intranasal challenge, both immunized groups were equally efficient in clearing *Bordetella pertussis* from the lungs. This study importantly shows that immunization with omvPV provides a milder inflammatory responses but with equal protection to bacterial colonization and induction of protective antibody and Th1/Th17 type immune responses compared to wPV. These results emphasize the potential of omvPV as a safe and effective next-generation pertussis vaccine.

The efficacy of pertussis vaccines on the market, whole-cell pertussis vaccine (wPV) and acellular pertussis vaccine (aPV), is under scrutiny because of a whooping cough resurgence despite high vaccination coverage^1^,^2^. The current view on immunity to *Bordetella pertussis* (*B. pertussis*) is that T-helper 1 and 17 (Th1/Th17) responses^3^,^4^ and specific antibody responses are preferred for protection. wPV induce a predominant Th1/Th17 response^4^,^5^,^6^ and a broad systemic antibody repertoire^7^, but are associated with mild adverse effects^8^,^9^. The suspected correlation between wPV and serious acute neurological illness in children^10^,^11^ has finally led to the call for safer pertussis vaccines. This resulted in the introduction of better-defined aPVs in many countries. aPVs evoke high IgG1 antibody titers and mainly a Th2 response, which provides protection against disease, however, with a relatively short duration. Recent findings for aPV suggest waning immunity in children^12^ and impaired prevention of transmission in baboons^13^. It is thought that aPV induce suboptimal antibody^7^ and T-cell responses^14^. These drawbacks call again for improved pertussis vaccines.

Outer membrane vesicles from *B. pertussis* (omvPV) are a potential vaccine candidate. The protection in mice induced by omvPV is comparable to that of aPV, based on lung colonization data after *B. pertussis* challenge^15^,^16^. Nevertheless, the omvPV elicits a broader humoral immunity and T-cell response compared to aPV^7^,^17^. To match the high short-term efficacy and good safety profile of current aPVs, a more comprehensive insight into omvPV-induced responses is required to unravel the type of immunity and assist in future vaccine registration. To that end, an unbiased and detailed systems biology approach is desirable. The application of systems biology in vaccine research has provided a better understanding of immune mechanisms and has been useful for prediction of vaccine efficacy based on correlating biomarkers both for yellow fever and influenza^18^,^19^,^20^. Moreover, systems biology can serve to compare molecular signatures induced by distinct vaccines^21^,^22^ and gain insight into vaccine safety^23^. Previously we used a systems approach in mice to study *B. pertussis* infection-induced responses^24^.

Here, we implemented systems vaccinology to investigate the potency of omvPV in mice. As benchmark, wPV was included for its relevant immune responses. Importantly also markers for vaccine safety with respect to pro- and anti-inflammatory cytokine secretion and splenic transcriptome were compared. Finally, the recall of protective immune responses in omvPV versus wPV immunized mice after *B. pertussis* challenge was compared and related to responses in non-protected mice.

## Methods

### Vaccines and challenge culture

OmvPV from *B. pertussis* B1917 were produced as previously described^25^. For preparation of wPV, *B. pertussis* B1917 was heat-inactivated (30 min, 56 °C) in PBS. Both omvPV and wPV were diluted in PBS to a final concentration of 4 μg total protein per immunization dose (300 μl). Vaccine characterization included particle size, protein composition, and LPS and DNA content^7^. For the challenge culture, stock suspension of *B. pertussis* strain B1917 was diluted in Verweij medium (BBio, Bilthoven, The Netherlands) to a final concentration of 5 × 10^6^ colony-forming units (cfu)/ml.

### Animal experiment

An independent ethical committee of the Institute for Translational Vaccinology (Intravacc) approved the animal experiment with identifier 201200073. Animal handling in this study was carried out in accordance with relevant Dutch national legislation, including the 1997 Dutch Act on Animal Experimentation. 8-week old female BALB/c mice (Harlan, The Netherlands) were immunized subcutaneously on day 0 (left groin) and day 28 (right groin) with 4 μg total protein of either omvPV or wPV resulting in omvPV-immunized mice (omvPV-mice) and wPV-immunized mice (wPV-mice). Mice were challenged intranasally under anesthesia (isoflurane/oxygen) with 2 × 10^5^ cfu *B. pertussis* B1917 in 40 μL Verweij medium on day 56. Non-immunized mice (N.I.-mice) were used as a control.

**Figure 1:**
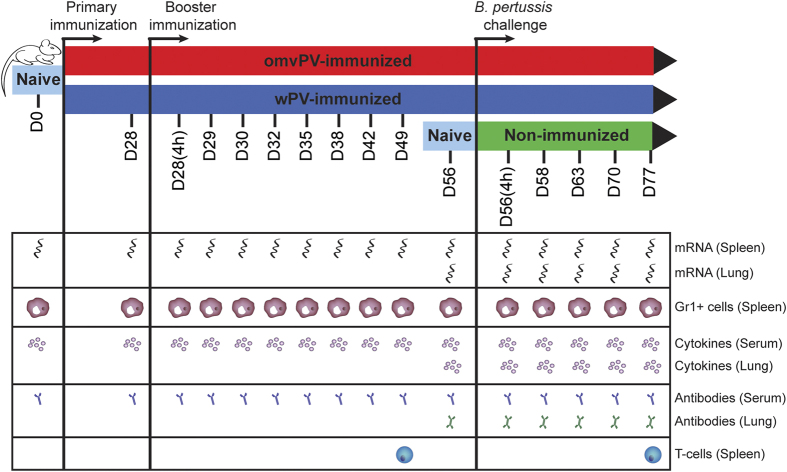
Study design of the systems approach on omvPV- and wPV-induced responses. BALB/c mice were subcutaneously immunized with 4 μg omvPV (red) or wPV (blue) on day 0 and day 28. Subsequently, the vaccine-induced responses of both vaccines were characterized over a period of 56 days at 10 different time points. Additionally, an intranasal *B. pertussis* challenge (2 × 10^5^ cfu/mouse) was performed on day 56 in immunized groups and in non-immunized mice (green). Both vaccine- and infection-induced responses were characterized at a transcriptomic, proteomic and cellular level on given time points as depicted.

For the determination of gene expression in spleen, cytokine responses and antibody responses, mice (n = 4) were sacrificed on day 28 after primary immunization. In addition, mice (n = 4) were euthanized after booster immunization on day 28 + 4 hours (day 28(4 h)), and day 29, 30, 32, 35, 38, 42, 49 and 56. Finally, mice (n = 4 per group) were sacrificed 4 hours after challenge and on day 58, 63, 70, and 77 to measure bacterial load in the respiratory tract, antibody responses and cytokine responses. For the investigation of Th subsets, mice (n = 4) were sacrificed on day 49 and 77. Naive mice (n = 4) were included as additional control group on each time point. Mice were bled under anesthesia (isoflurane/oxygen) by orbital bleeding and sacrificed by cervical dislocation for further sample collection. An overview of the study design is depicted in Fig. 1.

### Sample collection and preparation

For investigation of cytokine and antibody responses in serum, whole blood from each mouse was collected in a blood collection tube (MiniCollect 0.8 ml Z Serum Sep GOLD, Greiner Bio-One, Austria). After coagulation (10 min. at room temperature) and centrifugation (10 min., 3000 g), sera were taken and stored at −80 °C. For the colonization assay, the right lung lobe was placed in 900 ml Verweij medium at room temperature. To determine pulmonary cytokine and antibody responses, the lung lysates used for colonization assays were filtered (Millex GV Filter unit 0.22 μm, Millipore) and stored at −80 °C. For characterization of pulmonary gene expression, the left lung lobe was placed in 1 ml RNAlater (Qiagen), incubated overnight at 4 °C, and stored at −80 °C. In addition, the spleen was excised and divided in two equal parts. For gene expression analysis, one piece was placed in 1 ml RNAlater (Qiagen), incubated overnight at 4 °C and stored at −80 °C. For detection of Gr1^+^ cells, the other piece was placed in in 5 ml RPMI-1640 medium (Gibco) supplemented with 10% FCS (Hyclone), 100 units penicillin, 100 units streptomycin, and 2.92 mg/ml L-glutamine (Invitrogen), hereafter called RPMI complete medium, and kept on ice. For the analysis of Th responses, the whole spleen was placed in 5 ml of RPMI medium and kept on ice. Splenocytes were isolated by homogenization of spleens using a 70-μm cell strainer (BD Falcon, BD Biosciences) in RPMI complete medium.

### Colonization assay

The numbers of cfu in lung tissue were determined as previously described^24^.

### RNA isolation and microarray analysis

RNA isolation from lung and spleen tissue and a RNA quality check (concentration and integrity) were performed as described before^24^. Microarray analysis was performed on RNA concentrates from spleen tissue of each omvPV-mice and wPV-mice (n = 3) sacrificed at 16 time points: D0, D28, D28(4 h), D29, D30, D31, D32, D35, D42, D49, D56, D56(4 h), D58, D63, D70 and D77 (Fig. 1). For microarray analysis on lung tissue, RNA samples were analyzed obtained from individual mice sacrificed at time points: D56, D56(4 h), D58, D63, D70 and D77. RNA samples extracted from lung and spleen tissue of N.I.-mice were pooled (n = 3) for according time points described before. Amplification, labeling and hybridization of RNA samples for either lung or spleen tissue to microarray chips (NimbleGen 126135 k Mus musculus, Roche, Germany) was carried out at the Microarray Department of the University of Amsterdam, The Netherlands, as described previously^24^.

### Data analysis of gene expression

Quality control and normalization of raw microarray data was performed as described before^24^. To identify differentially expressed genes (DEGs) between experimental groups (naive and various time points post immunization or challenge) an ANOVA was applied. The induction or repression of individual genes was expressed as fold ratio (FR) by comparing mean gene expression levels of experimental groups to the naive mice. Average normalized gene expression levels contain data of three mice per immunized group. The data of challenged non-immunized mice are individual samples of pooled RNA of three mice. The dataset of challenged non-immunized mice (n = 3) of our previous study^24^ was incorporated in the analysis to increase the statistical power. Probes were considered differentially expressed if they met the following two criteria: (i) a *p*-value <0.001 (ANOVA), which corresponds to a Benjamini-Hochberg False discovery rate (FDR)^26^ of <5%; and (ii) an absolute FR ≥1.5 (experimental groups compared to naive mice) for at least one time point. If multiple probes corresponding to the same gene were differentially expressed, their data were averaged to remove redundancy for further analysis. GeneMaths XT (Applied Maths, St-Martens-Latem, Belgium) was used to visualize differences in gene expression in heatmaps and perform the hierarchical clustering based on Euclidean distance (linear scaling) with UPGMA. Genes were arranged according to similar expression patterns in time at which genes exceeded the FR cut-off of 1.5. To facilitate visual interpretation of heatmaps, only induction (red) and repression (green) of gene expression levels with fold ratios ≥1.5 are visualized, therefore presenting fold ratios ≤1.5 as naive level (black). Functional enrichment with an over-representation analysis (ORA) based on Gene Ontology Biological Processes (GO-BP) and Kyoto Encyclopedia of Genes and Genomes (KEGG) by using DAVID and the detection of cell-specific or tissue-specific gene expression based on BioGPS datasets was performed as described previously^24^. Involvement of type I and II IFN-signaling pathway was performed by using the Interferome database (http://www.interferome.org/interferome/home.jspx)^27^. Additional text mining on gene function was performed in PubMed.

### Multiplex immunoassay (MIA) and ELISA for antibody response

Levels of pulmonary IgA and serum IgM, IgA, total IgG and IgG subclasses (IgG1, IgG2a, IgG2b and IgG3) specific for *B. pertussis* antigens pertussis toxin (Ptx), filamentous hemagglutinin (FHA), pertactin (Prn), combined fimbria type 2 and 3 antigens (Fim2/3) and outer membrane vesicles B1917 (OMV B1917) were determined as described previously^24^ and data are presented in fluorescent intensity units (F.I.). Whole-cell *B. pertussis* ELISA to quantify total serum IgG was performed as described before^24^.

### Immunoproteomic profiling of serum IgG antibodies

Antigen specificity of *B. pertussis*-specific serum IgG antibody responses were determined by SDS-PAGE and subsequent Western blotting as described before^7^.

### Gr1^+^ cells in the spleen

The percentage of Gr1^+^ cells in the spleen was determined with flow cytometry as described before^24^.

### Isolation and *in vitro* restimulation of splenocytes

After treatment with erythrocyte lysis buffer, splenocytes were cultured in 24-well plates at 6 × 10^6^cells/well for 7 days at 37 °C in a humidified atmosphere containing 5% CO_2_ in IMDM medium (Gibco) supplemented with 8% FCS, 100 units penicillin, 100 units streptomycin, 2.92 mg/ml L-glutamine, and 20 μM β-mercaptoethanol (Sigma). The cells were either left unstimulated (medium control) or stimulated with 1 μg/ml Prn, 1 μg/ml Ptx, 1 μg/ml FHA, 5 μg/ml OMV, or 5 μg/ml heat-killed *B. pertussis*. On day 7, supernatant was collected for cytokine analysis.

### Cytokine profiling using multiplex technology

Concentrations (pg/ml) of 32 cytokines (Eotaxin, G-CSF, GM-CSF, IFNγ, IL-10, IL-12 (p40), IL-12 (p70), IL-13, IL-15, IL-17A, IL- 1α, IL-1β, IL-2, IL-3, IL-4, IL-5, IL-6, IL-7, IL-9, IP-10, KC, LIF, LIX, M-CSF, MCP-1, MIG, MIP-1α, MIP-1β, MIP-2, RANTES, TNFα, VEGF) present in serum and lung lysates were determined using a MIA (Milliplex MAP Mouse Cytokine/Chemokine - Premixed 32 Plex; Merck KGaA, Darmstadt, Germany) following the manufacturer’s protocol.

The concentration of various Th cytokines (IL-4, IL-5, IL-10, IL-13, IL-17A, TNFα, and IFNγ) was determined in culture supernatant using a Milliplex mouse cytokine 7-plex luminex kit (Millipore), according to the manufacturer’s protocol. Measurements and data analysis were performed with Bio-Plex 200, using Bio-PlexManager software (version 5.0, Bio-Rad Laboratories). Results were corrected for the background (medium control) and cytokine concentrations were calculated and provided in pg/ml.

### Statistical analysis

Data of the antibody, cytokine, and colonization assays were log-transformed after which a t-test was performed. *p*-values ≤ 0.05 were considered to indicate significant differences.

## Results

### OmvPV provides equal protection as wPV against *B. pertussis* challenge

**Figure 2:**
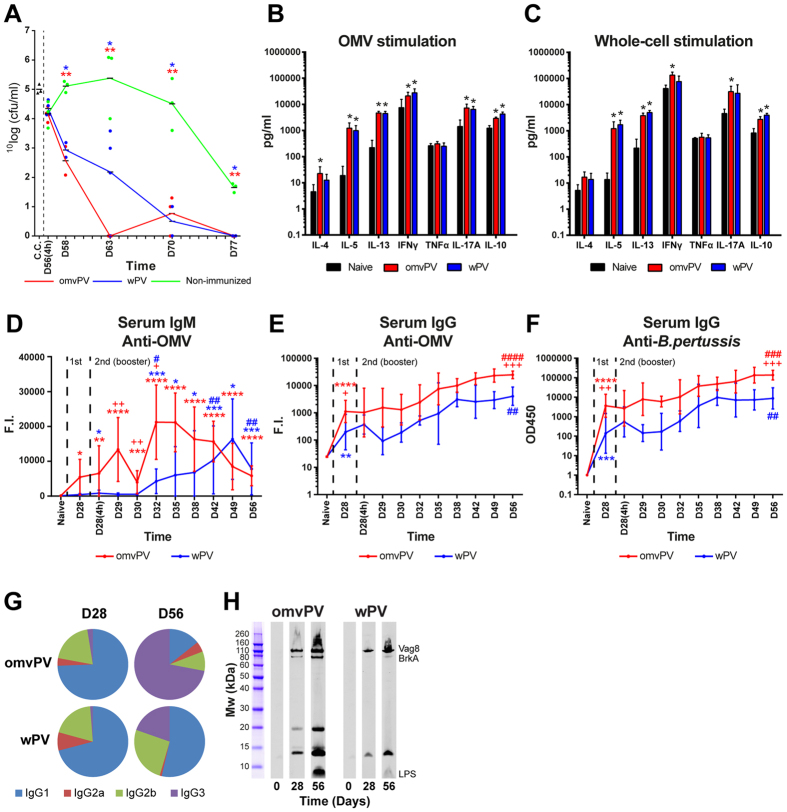
Lung colonization and vaccine-induced adaptive responses in omvPV- and wPV-immunized mice. (**A**) The number of *B. pertussis* cfu in 1 ml challenge culture (c.c.) was confirmed before challenge. Subsequently, the cfu/ml in the lungs of mice was determined 4 hours and 2, 7, 14, and 21 days after challenge (D56(4 h)-D77). * and **p ≤ 0.05 and *p* ≤ 0.01 for immunized group vs. non-immunized group. (**B,C**) Splenocytes were obtained post booster immunization (day 49) of mice that were naive (black), omvPV-immunized (red), or wPV-immunized (blue). Cytokine concentrations were determined in the culture supernatants after 7 days of stimulation with (**B**) 5 μg/ml OMVs or (**C**) 5 μg/ml *B. pertussis* whole-cells. Results for each mouse are corrected for medium stimulation. Data presented as mean ± SD (n = 4). *p ≤ 0.05 for immunized group vs. naive group. (**D–H**) The kinetics of serum (**D**) anti-OMV IgM, (**E**) anti-OMV IgG and (**F**) anti-*B. pertussis* IgG formation was determined for a period of 56 days after omvPV and wPV immunization. Data are presented as mean ± SD (n = 4). For (**D**) ^*^, ^**^, ^***^ and ****p ≤ 0.05, *p* ≤ 0.01, *p* ≤ 0.001 and *p* ≤ 0.0001 for day 28–56 vs. naive, ^#^ and ^##^*p* ≤ 0.05 and *p* ≤ 0.01, for D28(4 h)-D56 vs. D28, ^+^ and ^++^*p* ≤ 0.05 and *p* ≤ 0.01 for omvPV-mice vs. wPV-mice. For (**E,F**) ^*^, ^**^, ^***^ and ****p ≤ 0.05, *p* ≤ 0.01, *p* ≤ 0.001 and *p* ≤ 0.0001 for naive vs. D28, ^#^, ^##^, ^###^ and ^####^*p* ≤ 0.05, *p* ≤ 0.01, *p* ≤ 0.001 and *p* ≤ 0.0001 for D28 vs. D56, ^+^ and ^++^*p* ≤ 0.05 and *p* ≤ 0.01 for omvPV-mice vs. wPV-mice. The significance in (**E**) and (**F**) between D28(4 h)-D49 is not depicted. (**G**) Subclass distribution was determined at day 56 in serum of mice immunized with omvPV or wPV by calculating the ratio of the level for each subclass from the sum of the levels all subclasses. (**H**) To determine antigen-specificity, the immunoproteomic profiles of serum antibodies (pooled sera, n = 4) following primary immunization (day 28) and booster immunization (day 56) were obtained. The band positions of Vag8, BrkA and LPS antigens are indicated as identified previously^7^.

The composition of both omvPV and wPV, in terms of proteins, DNA, and LPS^7^, was determined before mice were immunized twice with a four-week interval (Fig. 1). Vaccine efficacy was assessed by determining lung colonization upon an intranasal *B. pertussis* challenge (Fig. 2A). N.I.-mice showed extensive colonization and lungs were not cleared at day 77 (21 days post challenge) as observed before^24^, whereas lungs of immunized mice were cleared faster. The clearance of lungs was not significantly different between omvPV-mice and wPV-mice, although the lungs of omvPV-mice tended to be cleared slightly faster than of wPV-mice.

### Immunization with omvPV and wPV induces a mixed Th1/Th2/Th17 response

Stimulation of splenocytes with *B. pertussis* OMVs or whole-cells resulted in enhanced production of IFNγ (Th1), IL-5 and IL-13 (Th2), IL-17A (Th17) and IL-10 in omvPV-mice and wPV-mice, compared to naive mice (Fig. 2B,C). TNFα was not observed, while IL-4 (Th2) was only detected after OMV stimulation in omvPV-mice. Hardly any cytokines were produced upon stimulation of splenocytes with the purified antigens Ptx, FHA and Prn, except for IL-17A after FHA stimulation in wPV-mice and IL-5 after Prn stimulation in omvPV-mice (Supplementary Fig. S1A–C, left panels).

### OmvPV induces higher serum IgG antibody responses

OMV-specific IgM was induced by omvPV and at a lower rate by wPV with highest peaks at day 32 and day 49, respectively (Fig. 2D). Moderate IgG levels were detected 28 days after the primary immunization with either omvPV or wPV, using an anti-OMV MIA and a whole-cell ELISA (Fig. 2E,F). Booster immunization strongly increased IgG production in omvPV-mice and wPV-mice, but omvPV induced higher IgG levels than wPV. No IgG or IgM antibodies directed against purified antigens (Ptx, FHA, Prn, Fim2/3) were found (data not shown). The IgG subclass distribution on day 28 was dominated by IgG1 for both omvPV and wPV (Fig. 2G). Booster immunizations with wPV and to a much larger extent with omvPV stimulated IgG3 antibody production as is shown on day 56 (Fig. 2G). Most of these IgG3 antibodies were directed against LPS^7^. Confirmed here, higher levels of anti-LPS IgG antibodies were detected in omvPV-mice compared to wPV-mice (Fig. 2H). Moreover, these anti-LPS IgG antibodies were observed solely on day 56, indicating that these antibodies were elicited by the booster immunization. Additionally, both vaccines mainly induced anti-Vag8 and anti-BrkA antibodies (Fig. 2H).

### Less pro-inflammatory cytokine induction in serum by omvPV compared to wPV

**Figure 3:**
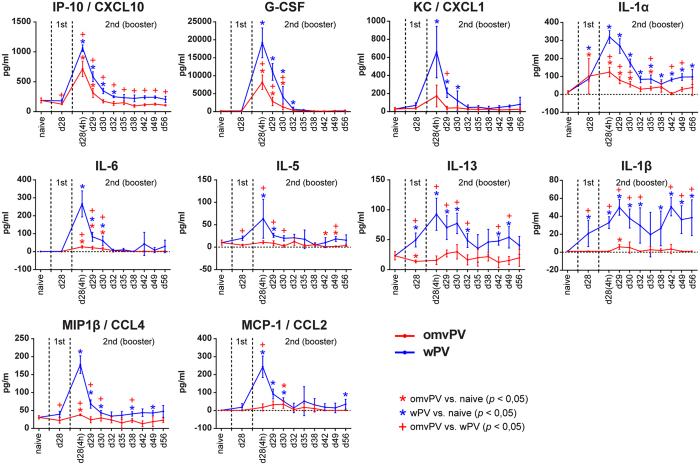
Serum cytokine secretion following omvPV or wPV immunization. Concentrations of 32 cytokines were determined in serum at multiple time points after immunization with omvPV or wPV. Levels of CXCL10, G-CSF, CXCL1, IL-1α, IL-6, IL-5, IL-13, IL-1β, CCL4, and CCL2 were significantly altered and are depicted here. The different stages of primary immunization and booster immunization are depicted in the panels. Data represent mean ± SD (n = 4). *p ≤ 0.05 for immunized group vs. naive, ^+^*p* ≤ 0.05 for omvPV group vs. wPV group.

Vaccine-induced cytokine responses in serum occurred mainly 4 hours after booster immunization (day 28(4 h)). Concentrations of CCL4, CXCL10, G-CSF, IL-1α, and IL-6 were significantly enhanced in omvPV-mice and wPV-mice compared to levels before immunization (Fig. 3). In omvPV-mice, all these cytokine concentrations were significantly lower than in wPV-mice. Moreover, CCL2, CXCL1, IL-1β, IL-5, and IL-13 concentrations were only significantly elicited in wPV-mice.

### Enhanced anti-inflammatory transcriptomic profiles in the spleen after omvPV immunization

**Figure 4:**
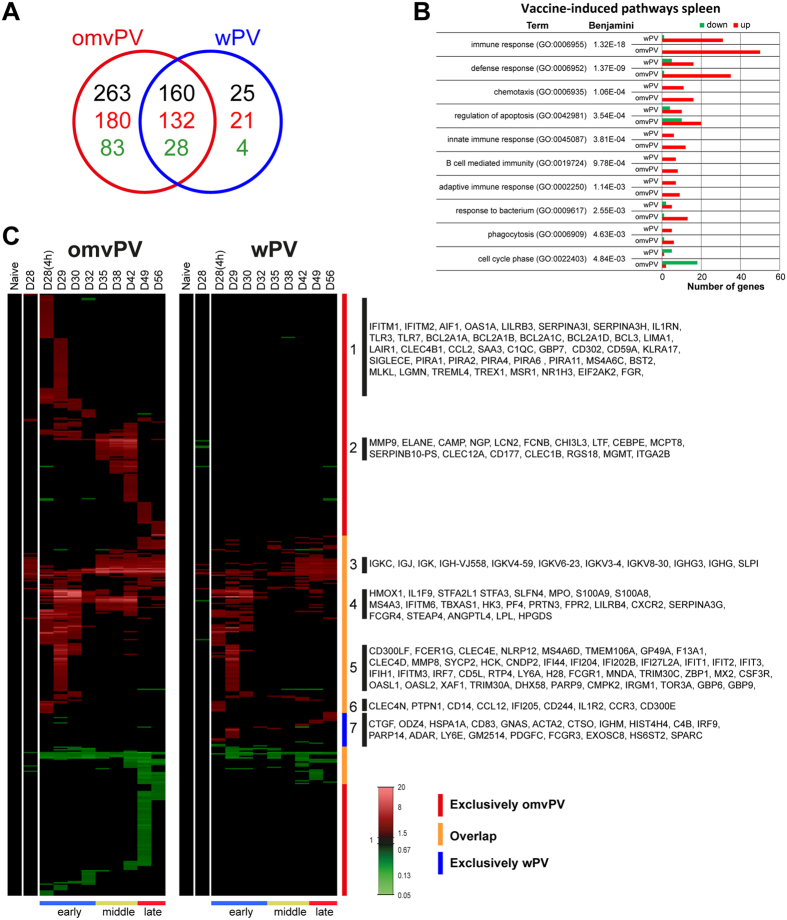
Transcriptomic profiles in the spleen following omvPV and wPV booster immunization. (**A**) Fold changes of DEGs were calculated compared to naive mice (FR ≥1.5, *p*-value ≤ 0.001). In total, 448 DEGs were found divided over both vaccine responses, as depicted in a Venn-diagram with total number of genes (black) and both upregulated (red) and downregulated genes (green). (**B**) Overrepresentation analysis on all 448 genes revealed the involvement of specific GO-BP terms and KEGG pathways with corresponding upregulated (red) and downregulated (green) genes. (**C**) All differentially upregulated (red) and downregulated (green) genes are portrayed as heatmap (mean of n = 4). Genes not surpassing a FR of 1.5 are shown as basal level (black) at this time point. Gene clustering is based on up/downregulation, time of involvement, and presence in the immunization group. The overlap and the exclusive presence of DEGs in either the omvPV or wPV groups are further depicted next to the heatmap. Booster immunization-induced responses were divided in three phases: early phase (D28(4 h)-D32), middle phase (D35-D42) and late phase (D49-D56) as calculated in Supplementary Fig. S2A. Selections of genes co-expressed in seven (1–7) areas of the heatmap are shown.

In total, 423 and 185 differentially expressed genes (DEGs) were identified in the spleen compared to naive mice (day 0) (*p*-value ≤ 0.001, FR ≥1.5) in omvPV-mice and wPV-mice, respectively (Fig. 4A). Of these DEGs, 160 (132 upregulated, 28 downregulated) overlapped between both immunized groups. Additionally, 263 DEGs (180 upregulated, 83 downregulated) were exclusively detected in omvPV-mice, whereas 25 DEGs (21 upregulated, 4 downregulated) were only found in wPV-mice.

Overrepresentation analysis (ORA) gained insight into molecular pathways activated by immunization and revealed enrichment of 77 gene ontology biological pathways (GO-BP) terms and 2 KEGG pathways (Benjamini ≤0.05) in the dataset of omvPV-mice and/or wPV-mice. The immunity-related pathways are shown in Fig. 4B. The terms *Immune response, Defense response,* and *Cell cycle* contained more genes upregulated in omvPV-mice than in wPV-mice. Both groups had an equal number of genes related to *Phagocytosis* and *B-cell mediated immunity* (Fig. 4B).

Genes were clustered based on (i) co-expression over time and (ii) overlap between both immunized groups (Fig. 4C). Additionally, the vaccine-induced response could be divided in three phases based on hierarchical clustering (Supplementary Fig. S2A): early phase (D28(4 h)-D32), middle phase (D35–D42) and late phase (D49–D56) (Fig. 4C). By gene clustering, 7 groups were identified and reviewed in-depth using text mining. The results are described in the following paragraphs.

Group 1 consists of DEGs that were exclusively expressed in the early phase of the omvPV-induced response. This group comprised genes related to pathogen recognition, such as *Fpr1* and genes related to Toll-like receptor (TLR) 3 (*Tlr3, Mlkl*) and TLR7 (*Tlr7, Treml4, Lgmn, Trex1*) mediated signaling. *Trex1* limits pro-inflammatory signals following TLR7 activation in macrophages^28^, whereas *Siglece* represses TLR-signaling in general^29^. Moreover, IL-1 receptor antagonist (*Il1rn*) and members of the Bcl-family, (*Bcl2a1a, Bcl2a1b, Bcl2a1c, Bcl2a1d, Bcl3*), paired-Ig-like receptors (*Pira1, Pira2, Pira4, Pira6, Pira11, Lilrb3*), and IFN-induced transmembrane proteins (*Ifitm1, Ifitm2*) were detected. Genes related to processing of LPS included *Bst2*, *Eif2ak2*, essential for LPS-induced iNOS production^30^, and *Msr1* that inhibits LPS-stimulated IL-6 secretion^31^.

Group 2 genes were exclusively upregulated in the middle phase of the omvPV-induced response. This group contained genes related to myeloid cells (*Mcpt8, Clec12a, Chi3l3*) and more specifically neutrophils (*Cd177, Cebpe, Clec1b*, *Elane, Lcn2, Ltf*, *Ngp*).

**Figure 5:**
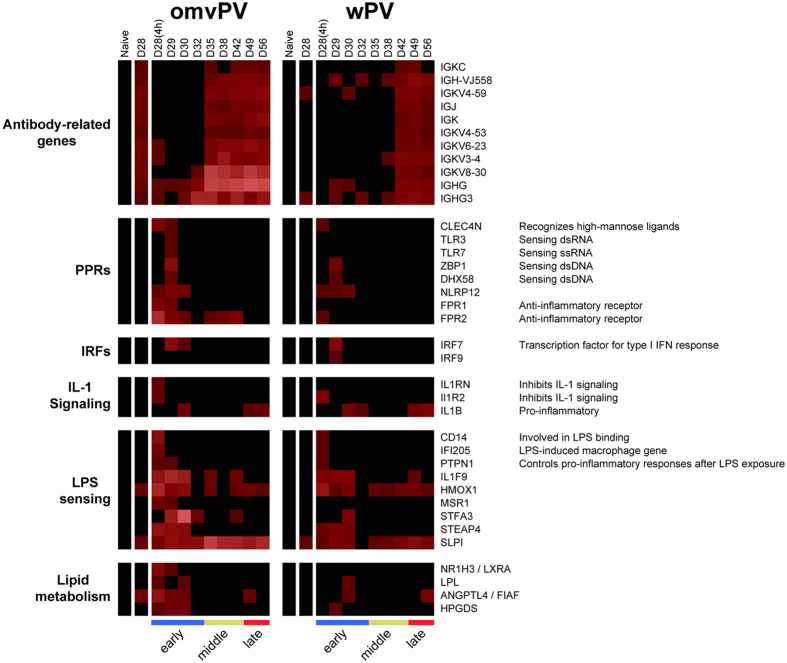
Selected splenic transcriptome profiles following omvPV and wPV booster immunization. The splenic transcriptome profiles of genes related to antibodies, pathogen recognition receptors (PRRs), interferon regulatory factors (IRFs), IL-1 signaling, LPS sensing, and lipid metabolism are depicted.

Group 3 includes antibody-related genes, such as *Igkc, Igj, Igk,* and *Ighg*, which showed higher expression in omvPV-mice than in wPV-mice (Fig. 5A), in line with the increased antibody levels (Fig. 2D–F).

Group 4 genes were upregulated in the early phase of immunization in omvPV-mice, but were not or hardly upregulated in wPV-mice. Most of these genes were also differentially expressed in the middle phase. This group contained the anti-inflammatory *Fpr2*^32^ and *Steap4* that decreases inflammatory effects by repressing IL-6 production^33^. Moreover, genes involved in LPS responsiveness were upregulated, such as *Slpi* that suppresses responses to LPS^34^, *Hmox1*, *Stfa3*, *Stfa2l1*, and *Angptl4* that are induced by LPS in the acute phase response^35^. Notably, *Angptl4* has multiple functions, including inflammation and lipid metabolism^36^. Genes involved in metabolism (*Hk3, Tbxas1*) and more specifically the lipid metabolism (*Angptl4, Lpl, Hpgds*) were found in this group. Furthermore, this group comprised *Pf4/Cxcl4*, myeloid cell specific genes (*Lilrb4, Ms4a3, Serpina3g*), danger-associated molecular patterns (DAMPs; *S100a9, S100a8*), IFN-induced genes (*Slfn4*, *Ifitm6*) and *Fcgr4* that binds IgG2a and IgG2b. Moreover, neutrophil-related genes (*Mpo*, *Prtn3*, *Cxcr2*, *Il1f9*) were detected. Notably, IL-1F9 promotes Th1 formation by binding IL-36R on T-cells^37^.

Group 5 contains genes that were expressed by both vaccines on day 29. Some of these genes are involved in the type I IFN-signaling pathway. This included *Irf7*, *Oasl1*, that inhibits IRF7^38^, and other IFN-induced proteins (*Ifi44, Ifi204, Ifi202b, Ifi27l2a, Ifit1, Ifit2, Ifit3, Ifih1, Ifitm3, Irgm1, H28, Mnda, Oasl2, Tor3a, Lgals3bp, Mx2*). Moreover, two genes were detected that encode pathogen recognition receptors (PRRs) sensing cytosolic DNA: DAI (*Zbp1*)^39^ and LPG2 (*Dhx58*)^40^. Furthermore, genes encoding Fc receptors (*Fcgr1, Fcer1g*), a NOD-like receptor and neutrophil migration marker^41^ (*Nlrp12*), C-type lectins (*Clec4d, Clec4e*), and other membrane markers (*Ly6a, Ms4a6d, Cd300lf, Gp49a*) were identified. Moreover, differential gene expression was detected for two genes encoding tripartite-motif proteins (*Trim30a, Trim30c*) of which TRIM30α inhibits TLR4-mediated NF-kappaB activation^42^.

Group 6 genes were upregulated on day 28(4 h) by both vaccines. This group included an LPS-induced macrophage gene (*Ifi205*), the inflammatory chemokine *Ccl12,* the interleukin 1 receptor, type II (*Il1r2*), and *Ptpn1* that controls pro-inflammatory responses after LPS exposure^43^. Moreover, genes encoding membrane markers were detected (*Cd244, Cd300e*, *Ccr3*) of which some are related to pathogen-recognition, such as dectin-2 (*Clec4n*) that recognizes high-mannose ligands, and *Cd14* involved in LPS binding.

Group 7 genes were merely differentially expressed in the wPV-mice. This group included genes on immune cells such as *C4b, Cd83* an important activation marker on B-cell, T-cell, and dendritic cell populations^44^, *Fcgr3*, *Ighm, Irf9*, *Ly6e*, and *Parp14*. PARP-14 promotes Th2 and Th17 cell formation and steers the antibody subclass distribution^45^,^46^.

Based on this text mining, sets of genes involved in antibody formation, PRRs, interferon regulatory factors (IRFs), IL-1 signaling, LPS sensing and lipid metabolism were found (Fig. 5). Subsequently, the dataset was matched with the Interferome database to identify which vaccine-induced genes were involved in the type I and type II IFN-signaling pathways (Supplementary Fig. S3). In total, 91 genes were related to type I IFN, 22 to type II IFN, and 60 genes to both pathways. The majority of IFN-related genes of both type I and II were induced by omvPV, whereas only 13 genes were found exclusively in wPV-mice. Moreover, the type II IFN-signaling pathway was mainly involved in the early phase. The type I IFN-signaling pathway genes were also mainly found in the early phase, but also partly in later phases (Supplementary Fig. S3).

### Higher neutrophil responses elicited by omvPV compared to wPV

Transcriptome comparison with BioGPS databases particularly revealed involvement of MAC^+^GR1^+^ granulocytes (neutrophils) in vaccine-induced immune responses (Supplementary Fig. S4A). These 46 genes were all detected in omvPV-mice and mainly activated during the early and middle phase of the vaccine-induced response (Supplementary Fig. S4B). In contrast, only 16 of these 46 genes were upregulated in wPV-mice. Flow cytometry confirmed that the percentage of Gr1^+^ cells in the spleen was significantly increased by omvPV and wPV immunization between days 29–42 (Supplementary Fig. S4C). In line with the enhanced gene expression, a significant higher number of Gr1^+^ cells was detected in the middle phase (day 42) in omvPV-mice compared to wPV-mice.

### Immunized mice showed reduced serum and pulmonary cytokine responses following *B. pertussis* challenge

**Figure 6:**
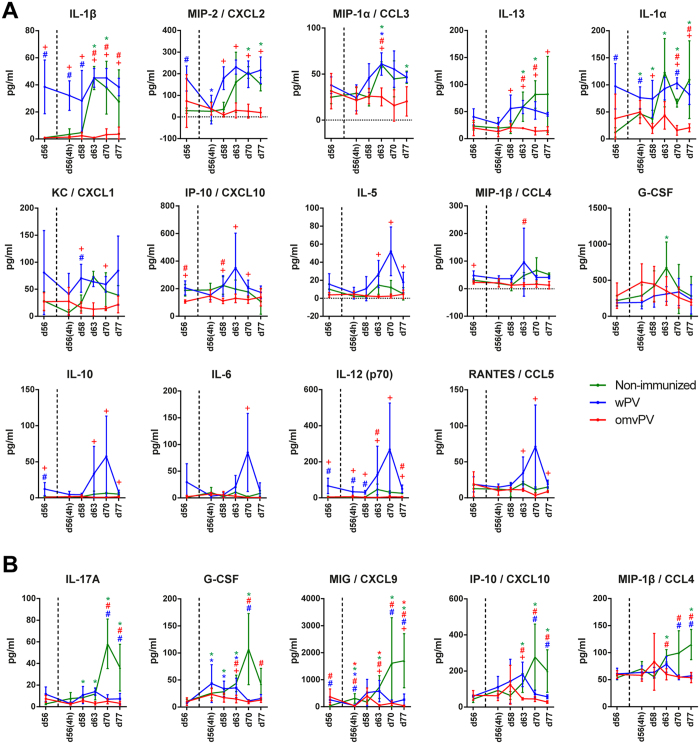
Serum and pulmonary cytokine secretion following *B. pertussis* challenge in mice immunized with omvPV or wPV and non-immunized mice. (**A,B**) The concentrations of 32 cytokines were determined prior to and at multiple time points post challenge with *B. pertussis* in non-immunized mice (green) or mice immunized with omvPV (red) or wPV (blue). Only cytokines that were significantly altered in (**A**) serum and (**B**) lung lysate are depicted. Data are presented as mean ± SD for immunized groups (n = 4) and non-immunized groups (n = 3). *p ≤ 0.05 for challenged groups vs. day 56, ^#^*p* ≤ 0.05 for immunized groups vs. non-immunized group, ^+^*p* ≤ 0.05 for omvPV group vs. wPV group.

Subsequent to the investigation of vaccine-induced responses, we examined the immune responses after *B. pertussis* challenge (Fig. 1). First, concentrations of 32 cytokines were determined in serum and lung lysate (Fig. 6A,B). In sera of N.I.-mice, IL-1α, IL-1β, CXCL2, CCL3, IL-13, and G-CSF were significantly increased mainly between days 63–77. Notably, no significant serum cytokine secretion was observed in omvPV-mice (Fig. 6A). Serum concentrations of IL-1α, IL-1β, IL-5, IL-6, IL-10, IL-12(p70), IL-13, CCL3, CCL4, CCL5, CXCL1, CXCL2, and CXCL10 were significantly higher in wPV-mice compared to omvPV-mice, mainly on day 63. However, the *B. pertussis* challenge did not lead to significant induction of cytokine levels compared to day 56 in wPV-mice, except for CCL3 (Fig. 6A). In the lungs, IL-17A, G-CSF, CXCL9, CCL4, and CXCL10 were significantly increased in N.I.-mice, between day 63–77 whereas these cytokines were not elevated in either of the immunized groups, except for G-CSF in wPV-mice between day 56(4 h)-63 (Fig. 6B).

### Reduced transcriptomic profiles in the spleen of immunized mice following *B. pertussis* challenge

**Figure 7:**
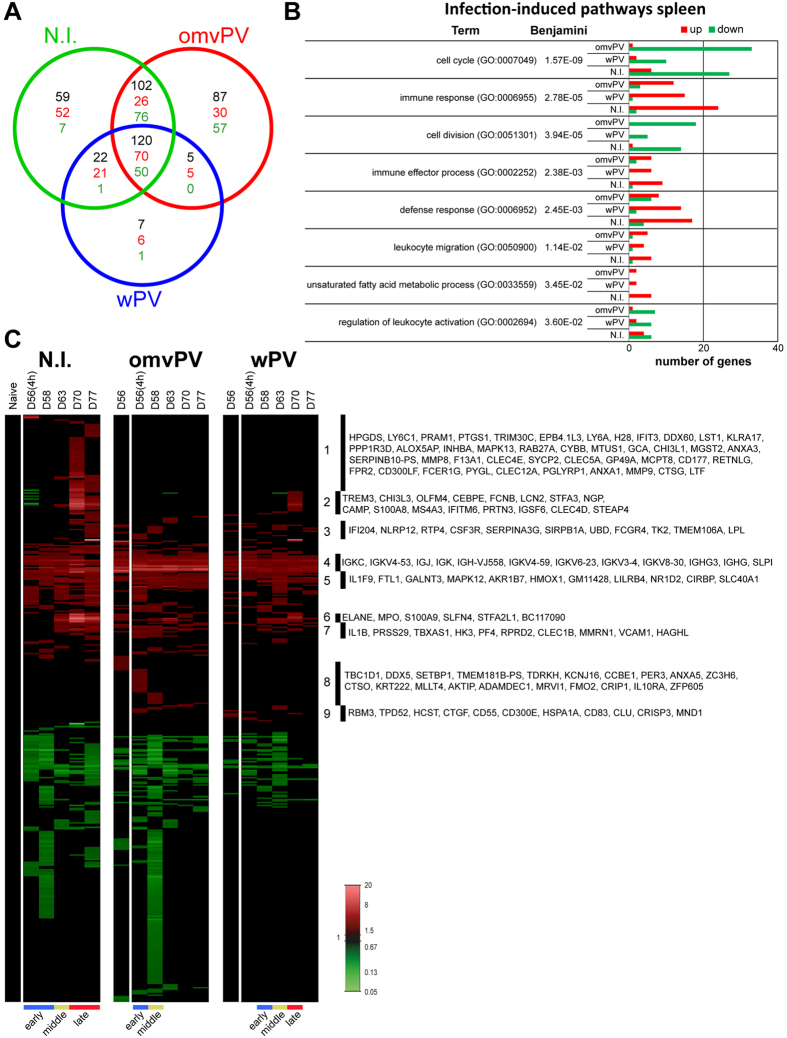
Transcriptomic profiles in the spleen following *B. pertussis* challenge in omvPV-, wPV-, and non-immunized mice. (**A**) Fold changes in expression and significant gene expression were calculated compared to naive mice (FR ≥1.5, *p*-value ≤ 0.001). In total, 402 DEGs were found divided over the three groups in a Venn-diagram with total number of genes (black), upregulated genes (red), and downregulated genes (green). (**B**) Overrepresentation analysis on all 402 genes revealed the involvement of specific GO-BP terms and KEGG pathways with corresponding upregulated (red) and downregulated (green) genes. (**C**) All differentially upregulated (red) and downregulated (green) genes are portrayed as heatmap (mean of n = 3 for immunized groups, n = 1 (pool of 3 mice for non-immunized group)). Genes not surpassing a FR of 1.5 are shown as basal level (black). Gene clustering is based on up/downregulation, time of involvement, and presence in different groups. Infection-induced responses were divided in phases according to the hierarchical clustering calculated in Supplementary Fig. S2B. Selections of genes in nine (1–9) areas of the heatmap are shown.

In total, 402 DEGs (*p*-value ≤ 0.001, FR ≥1.5) were detected in the spleen of immunized mice or N.I.-mice after *B. pertussis* challenge (Fig. 7A). This analysis demonstrated less intense gene expression profiles in omvPV-mice and wPV-mice than in N.I.-mice following the challenge with *B. pertussis*. These responses were even lower in omvPV-mice compared to wPV-mice. 87, 7, and 59 DEGs were exclusively detected in the challenged omvPV-mice, wPV-mice, and N.I.-mice, respectively. 120 DEGs (70 upregulated, 50 downregulated) were found in all three groups (Fig. 7A). The ORA on infection-induced responses in the spleen revealed enrichment of 24 GO-BP terms and 2 KEGG pathways (Benjamini ≤0.05) in omvPV-mice, wPV-mice, and N.I.-mice (Fig. 7B). In omvPV-mice, several downregulated genes are involved in *Cell cycle* (33 genes) and *Cell division* (18 genes). The terms *Immune response* and *Defense response* comprised a smaller number of genes in omvPV-mice than in wPV-mice and N.I.-mice. Genes were clustered based on (i) co-expression over time and (ii) overlap between both experimental groups (Fig. 7C). Hierarchical clustering on the three challenged groups indicated that infection-induced responses were distinct in immunized mice compared to the challenged N.I.-mice (Supplementary Fig. S2B). The infection-induced response of N.I.-mice was divided in three phases: early phase (D56(4 h)-D58), middle phase (D63), and late phase (D70–D77). In omvPV-mice, the response was faster compared to N.I.-mice. In wPV-mice, the response was slightly slower compared to omvPV (Fig. 7C and Supplementary Fig. S2B). A selection of genes involved in nine (1–9) areas is depicted in the heatmap. The involvement of neutrophils was more pronounced in challenged N.I.-mice as we detected 38 genes commonly expressed in MAC^+^GR1^+^ granulocytes compared to only 10 and 22 of these genes in omvPV-mice and wPV-mice, respectively (Supplementary Fig. S4D). Flow cytometry analysis confirmed a significantly increased number of Gr1^+^ cells, indicative for neutrophils, in N.I.-mice on day 70 (Supplementary Fig. S4E). This Gr1^+^ cell fraction in immunized mice was significantly lower between days 70–77 compared to N.I.-mice.

### *B. pertussis* challenge has less impact on gene expression in the lungs of omvPV-mice than in wPV-mice and N.I.-mice

In total, 3269 DEGs (*p*-value ≤ 0.001, FR ≥1.5) were detected in lungs of immunized mice or N.I.-mice following *B. pertussis* challenge (Supplementary Fig. S5A). The pulmonary transcriptome in omvPV-mice on day 56 prior to challenge differs a lot from the naive basal gene expression level. Compared to day 56, the *B. pertussis* challenge induced less change in pulmonary gene expression in omvPV-mice as compared to wPV-mice and N.I.-mice. Exclusive expression of 578, 156, and 90 DEGs was detected in challenged omvPV-mice, wPV-mice, and N.I.-mice, respectively. In total, 1702 DEGs (695 upregulated, 1007 downregulated) were found in all three groups (Supplementary Fig. S5A). Genes were clustered based on (i) co-expression over time and (ii) overlap between both experimental groups (Supplementary Fig. S5B). Hierarchical clustering of the pulmonary transcriptome of the three challenged groups demonstrated a distinct infection-induced response in immunized mice compared to challenged N.I.-mice (Fig. S2C). The infection-induced response of N.I.-mice was divided in three phases: early phase (D56(4 h)-D58), middle phase (D63 and D77) and late phase (D70), as seen before^24^. In omvPV-mice, the response was less diverse compared to day 56 and divided in two phases; early phase (D56(4 h)) and middle phase (D70). In wPV-mice, the response compared to day 56 was more diverse and developed slower than in omvPV-mice with an early phase (D63–D70) and middle phase (D77) (Supplementay Figs S2C and S5B).

### Adaptive recall responses in omvPV-mice and wPV-mice following *B. pertussis* challenge

**Figure 8:**
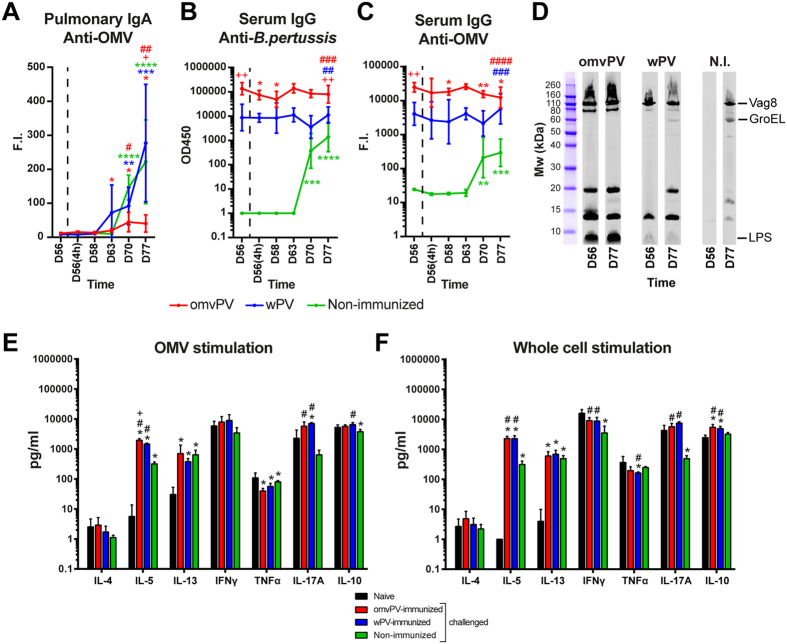
Humoral and cellular adaptive recall responses following *B. pertussis* challenge in omvPV- and wPV-immunized mice as compared to control mice before and after challenge. (**A–C**) Kinetics of (**A**) pulmonary anti-OMV IgA, (**B**) serum anti-*B. pertussis* IgG, and (**C**) serum anti-OMV IgG levels were determined for a period of 21 days following intranasal challenge in immunized mice (n = 4) and non-immunized mice (N.I.) (n = 3). Data are presented as mean ± SD. Statistical significance of differences in (**A**), (**B**) and (**C**): ^*^,^ **^, ^***^ and ****p ≤ 0.05, *p* ≤ 0.01, *p* ≤ 0.001 and *p* ≤ 0.0001 for vs. D56 per group, ^+^ and ^++^*p* ≤ 0.05 and *p* ≤ 0.01 for omvPV vs. wPV, ^#^, ^##^, ^###^ and ^####^*p* ≤ 0.05, *p* ≤ 0.01, *p* ≤ 0.001 and *p* ≤ 0.0001 for immunized groups vs. non-immunized group. (**D**) Immunoproteomic profiles of serum IgG antibodies (pooled sera, n = 4) before (D56) and 21 days post challenge (D77) were obtained on a *B. pertussis* lysate. (**E,F**) Cytokine concentrations were determined in the culture supernatants after 7 day stimulation with (**E**) 5 μg/ml OMVs, or (**F**) 5 μg/ml *B. pertussis* whole-cells. Splenocytes were obtained from naive mice (black) and post-challenge (D77) of mice that were omvPV- (red), wPV- (blue) or non-immunized (green). Results for each mouse are corrected for medium stimulation. Data in (**E**) and (**F**) presented as mean ± SD (n = 4). Statistical significance of differences in (**E**) and (**F**) *p ≤ 0.05 for challenged groups vs. naive, ^+^*p* ≤ 0.05 for omvPV group vs. wPV group, ^#^*p* ≤ 0.05 for immunized group vs. non-immunized group.

Prior to the intranasal *B. pertussis* challenge (day 56), no vaccine-induced anti-OMV IgA was detected in the lungs of omvPV-mice and wPV-mice. However, similar amounts of anti-OMV IgA were detected after the challenge of wPV-mice and N.I.-mice on day 63–77, but no anti-OMV IgA was found in omvPV-mice (Fig. 8A). Serum IgG levels specific for pertussis OMVs or whole-cells were unaltered in wPV-mice and slightly decreased in omvPV-mice after the challenge, whereas IgG levels in N.I.-mice were rising between day 63–77 (Fig. 8B,C). The IgG antibody specificity did not change in omvPV-mice after challenge, except for the disappearance of the 15 kDa band (Fig. 8D). The challenge of wPV-mice stimulated antibody formation against GroEL (60 kDa) and an unknown antigen of 20 kDa.

Regarding post challenge Th-responses, the stimulation of splenocytes with OMVs revealed altered Th1 (TNFα), Th2 (IL-5, IL-13), and Th17 (IL-17A) cytokines in challenged omvPV-mice, wPV-mice, and N.I.-mice compared to naive mice (Fig. 8E,F and Supplementary Fig. S1). IL-5 and IL-17A levels were higher in immunized mice compared to N.I.-mice (Fig. 8E). Stimulation with *B. pertussis* whole-cells led to altered levels of Th1 (IFNγ), Th2 (IL-5, IL-13) and Th17 (IL-17A) cytokines in omvPV-mice, wPV-mice, and N.I.-mice compared to naive mice. These levels were overall higher in immunized mice compared to N.I.-mice. Moreover, IL-10 production was only observed in immunized mice (Fig. 8F). IFNγ and TNFα were both high in naive control mice after stimulation with *B. pertussis* OMVs and whole-cells, most likely caused by LPS. Nevertheless, omvPV-mice and wPV-mice showed a similar result post challenge (day 77) as after immunization (day 49). Prn stimulation resulted in increased IL-13 and IL-17A concentrations in all challenged mice compared to naive control mice (Supplementary Fig. S1A, right panel). These IL-17A levels were significantly higher in immunized mice compared to N.I.-mice. Furthermore, significantly higher IL-5 production was observed in omvPV-mice and N.I.-mice than in wPV-mice. After Ptx stimulation, IL-17A production was exclusively detected in wPV-mice (Supplementary Fig. S1B, right panel). IL-5, IL-13 and IL-17A production was evoked by FHA stimulation in immunized and N.I.-mice. Moreover, enhancement of IFNγ, TNFα, and IL-10 was detected in wPV-mice (Supplementary Fig. S1C, right panel). Overall enhanced Th1/Th2/Th17 *ex vivo* recall responses were detected in challenged omvPV-mice and wPV-mice compared to challenged N.I.-mice.

## Discussion

Introduction of an improved next-generation pertussis vaccine requires in-depth knowledge on vaccine-induced responses in comparison to current pertussis vaccines especially in terms of efficacy and safety. Parenteral injection of classical whole-cell pertussis vaccine (wPV) has been related to adverse effects^8^,^9^ and suspected of being correlated with serious acute neurological illness in children^10^,^11^ mainly caused by circulating pro-inflammatory cytokines such as IL-1β, TNFα, and IL-6^11^,^47^. The adverse effects have been key reasons for the development of aPVs, which have a better safety profile. Our current *in vivo* study demonstrated that immunization with an OMV-based pertussis vaccine (omvPV) in comparison to wPV elicited reduced concentrations of serum pro-inflammatory cytokines (IL-1α, IL-1β, and IL-6), chemokines (CXCL1 and CXCL10), and G-CSF. Especially, reduced IL-1β secretion by omvPV may be beneficial, because IL-1β is related to acute neurological illness^11^. Although data obtained in mice may not be representative for humans, it was previously demonstrated with a human whole-blood stimulation assay that omvPV induces less IL-6 compared to wPV^17^, which is in line with the mouse data presented here. Other cytokines that were detected in the current *in vivo* mouse model could be investigated in a similar way or in future human clinical trials with omvPV. Activation of Toll-like receptors (TLRs)^48^ by pathogen-associated molecular patterns (PAMPs), like lipopolysaccharide (LPS) and bacterial DNA, result in the secretion of these cytokines. Previously we showed that omvPV contain less TLR-activating LPS (1.0 μg *versus* 1.5 μg) and DNA (0.2 μg *versus* 1.2 μg) than wPV^7^. Lower LPS and DNA concentrations in omvPV may induce less TLR4 and TLR9 activation and subsequently contribute to attenuated pro-inflammatory cytokine response by omvPV compared to wPV. Adsorption of LPS to aluminum hydroxide causes less pro-inflammatory cytokine production^49^, which demonstrates that free LPS might be more pyrogenic than bound LPS. OmvPV may contain less free LPS compared to wPV thus resulting in lower cytokine induction.

Unexpectedly, in light of the attenuated pro-inflammatory cytokine signatures, omvPV immunization induced a larger number of genes than wPV. Yet triggering of anti-inflammatory responses, diminished PRR responses, and expression of modifiers that dampen inflammatory LPS responses was more prominent in omvPV-mice than in wPV-mice and likely explains why omvPV elicited lower amounts of pro-inflammatory cytokines. The omvPV-activated genes encoding anti-inflammatory PRRs FPR1 and FPR2 that bind many different signal peptides of bacteria^50^ and are important for rapid neutrophil recruitment^51^. Furthermore, the involvement of omvPV-induced genes encoding FPR2, SPLI, STEAP4, PTPN1, and MSR1, which are attenuators of LPS responses^31^,^32^,^33^,^34^,^43^, may assist in repressing LPS-induced pro-inflammation. The more prominent anti-inflammatory response following omvPV immunization was strengthened by activation of genes encoding proteins with a dual function in lipid metabolism and anti-inflammation, namely ANGPTL4^35^,^36^, HPGD2S^52^, and LXRα. These genes may have been activated by lipid mediators that were released during the omvPV-induced response, which needs to be further investigated.

Only omvPV immunization enhances gene expression of TLR3 and TLR7, which are intracellular sensors for viral and bacterial nucleotides. Activation of TLRs leads to induction of IFN-signaling pathways. For instance, IRF7, a factor downstream of TLR7 and important mediator of type I IFN-signaling^53^, was induced by both vaccines. Overall, a large number of genes of the type I IFN-signaling and type II IFN-signaling pathway were induced by both vaccines but were more profound in the omvPV response. Type I IFN-signaling can affect both innate and adaptive immune cells^54^ and with respect to CD4^+^ T-cells, the pathway is important for the regulation of Th1 and Th17 responses^55^. Whether there are more TLR3 and TLR7 ligands present in omvPV compared to wPV remains to be investigated.

In addition, TLR4 activation by LPS can direct the cellular responses towards Th1 and Th17 responses^5^,^6^. Thus, it is important to evaluate whether the reduced TLR4-signaling by omvPV does not affect its ability to induce *B. pertussis* specific Th1 and Th17 responses. Cytokine profiles of stimulated splenocytes revealed that omvPV and wPV both induce mixed systemic Th1/Th2/Th17 responses, in contrast to the Th2-biased aPV-induced response^4^,^14^. This indicates that despite lower LPS concentration and TLR4-signaling, omvPV still induced a similar Th response as wPV. This may suggest that the LPS concentration in omvPV is sufficient for the induction of Th1/Th17 responses or that other signaling pathways, such as via TLR2 through lipoproteins^56^,^57^, also promote Th17 responses. Induction of both types of IFN-signaling pathways by omvPV and wPV may contribute to these Th1/Th17 responses that are thought to be important for protection^3^,^4^,^24^.

The omvPV and wPV used in this study contain low concentrations of aPV components (Prn, FHA, and Ptx). except for FHA in wPV^7^, which also resulted in low antibody and T-cell responses specific for these antigens following immunization. However, high antibody levels against other *B. pertussis*-specific antigens such as BrkA, Vag8 and LPS, were detected. These antibodies were especially formed after booster immunization and overall higher in omvPV-mice. The omvPV also contains higher concentrations of BrkA and Vag8^7^. As Prn-deficient *B. pertussis* strains are increasingly circulating, especially in aPV-immunized populations, it has been hypothesized that this might be the result of pathogen adaptation^58^,^59^. Therefore, the broad humoral response conferred by both omvPV and wPV might limit the risk of driving *B. pertussis* adaptation caused by vaccine-induced selection pressure. Remarkably, despite lower LPS concentrations in omvPV, the booster omvPV immunization induced higher anti-LPS IgG3 antibody levels than wPV^7^. This indicates that the LPS in omvPV was processed in a different way by the immune system during the first immunization compared to booster immunization. Since anti-LPS IgG3 antibodies are mostly produced in a T-cell independent manner^60^, this may indicate that omvPV evokes a more efficient T-cell independent B-cell response than wPV. Furthermore, mucosal *B. pertussis*-specific IgA induction in the lungs was absent after omvPV and wPV immunization, as expected. Mucosal IgA is produced after natural infection^24^ and contributes most likely to a faster lung clearance during *B. pertussis* challenge. Presumably, direct involvement of the respiratory tract through mucosal vaccine administration may result in more effective immunity.

Immunization with omvPV or wPV enabled rapid clearance of *B. pertussis* from the lungs after challenge when compared to N.I.-mice. Whereas no significant differences in lung clearance were observed between both immunized groups, the lungs of omvPV-mice tended to be cleared slightly faster perhaps because of the higher serum IgG levels. Previously, intraperitoneal immunization with alum-adjuvated omvPV and wPV derived from a *B. pertussis* Tohama strain also demonstrated equal protection^15^. The *B. pertussis* challenge elucidated the pre-existing immunity in omvPV-mice and wPV-mice. The immune responses of challenged immunized mice were characterized by (i) adaptive recall responses, (ii) a change in pulmonary environment by reduced cytokine secretion and transcriptome expression, (iii) altered systemic cytokine responses, and (iv) reduced splenic transcriptome expression in comparison to challenged N.I.-mice. The presence of high serum IgG levels and strong recall of Th1/Th2/Th17 responses enabled a rapid clearance in both immunized groups. In contrast to vaccine-induced responses, the challenge-induced responses revealed specific T-cell activation against purified proteins FHA, Prn and Ptx as a result of prolonged exposure to these antigens after *B. pertussis* challenge^24^. Furthermore, immunized mice responded less vigorously to an infection with respect to transcriptome, cytokine responses and number of splenic neutrophils. In line with our previous study^24^, N.I.-mice showed the largest changes in gene expression in the lungs 14 days after challenge, whereas these responses were overall lower in omvPV-mice than wPV-mice. Overall, *B. pertussis* challenge-induced responses in wPV-mice resembled those of N.I.-mice more than those of omvPV-mice, which may indicate that the *B. pertussis* challenge was controlled more effectively in omvPV-mice than wPV-mice. For instance, challenged omvPV-mice revealed no presence of antigen-specific IgA in the lungs nor IL-17A secretion after *ex vivo* Ptx stimulation of splenocytes, while these responses appeared in both wPV-mice and N.I.-mice. Therefore, the *B. pertussis* exposure in wPV-mice may have been longer than in omvPV-mice as this Ptx exposure only occurs during *B. pertussis* colonization, since Ptx is absent in both omvPV and wPV used in this study^7^.

In summary, we demonstrated that in comparison to a classical wPV, omvPV confers equal, if not more rapid protection with higher IgG levels and a comparable Th1/Th2/Th17 response. Additionally, the systems approach provided detailed insight into the molecular signatures of the vaccine as well as challenge-induced responses. Importantly, the inflammatory responses elicited by omvPV were milder than those by wPV, as reflected by reduced levels of pro-inflammatory cytokines. Probably, inflammatory responses are attenuated by the enhanced anti-inflammatory responses, i.e. ANGPTL4, FPR2, PTPN1, SPLI, and STEAP4. Therefore, it is tempting to speculate that the decreased inflammatory responses induced by omvPV reflect a better safety profile. In conclusion, our collective findings emphasize the potential of omvPV as a third-generation pertussis vaccine.

## Additional Information

**How to cite this article**: Raeven, R. H. M. *et al*. *Bordetella pertussis* outer membrane vesicle vaccine confers equal efficacy in mice with milder inflammatory responses compared to a whole-cell vaccine. *Sci. Rep.* **6**, 38240; doi: 10.1038/srep38240 (2016).

**Publisher’s note:** Springer Nature remains neutral with regard to jurisdictional claims in published maps and institutional affiliations.

## Supplementary Material

Supplementary figures S1–S5
