# Supplementary material for: Bordetella pertussis outer membrane vesicle vaccine confers equal efficacy in mice with milder inflammatory responses compared to a whole-cell vaccine
Source: Sci Rep. 2016 Dec 1;6:38240. doi: 10.1038/srep38240 (PMC5131296; doi:10.1038/srep38240)
Supplement: Supplementary figures S1–S5 [file srep38240-s1.pdf]

Supplementary Figures

as part of the manuscript

Bordetella pertussis outer membrane vesicle vaccine confers equal efficacy in mice with milder inflammatory responses compared to a whole-cell vaccine

René H. M. Raeven[1,2], Jolanda Brummelman[3], Jeroen L. A. Pennings[4], Larissa van der Maas[1], Wichard Tilstra[1], Kina Helm[3], Elly van Riet[1], Wim Jiskoot[2], Cécile A. C. M. van Els[3], Wanda G. H. Han[3], Gideon F. A. Kersten[1,2], Bernard Metz[1]*

[1] *Institute for Translational Vaccinology (Intravacc), Bilthoven, The Netherlands,*
[2] *Division of Drug Delivery Technology, Leiden Academic Centre for Drug Research, Leiden, The Netherlands,*
[3] *Centre for Infectious Disease Control, National Institute for Public Health and the Environment (RIVM), Bilthoven, The Netherlands,*
[4] *Centre for Health Protection (GZB), National Institute for Public Health and the Environment (RIVM), Bilthoven, The Netherlands*

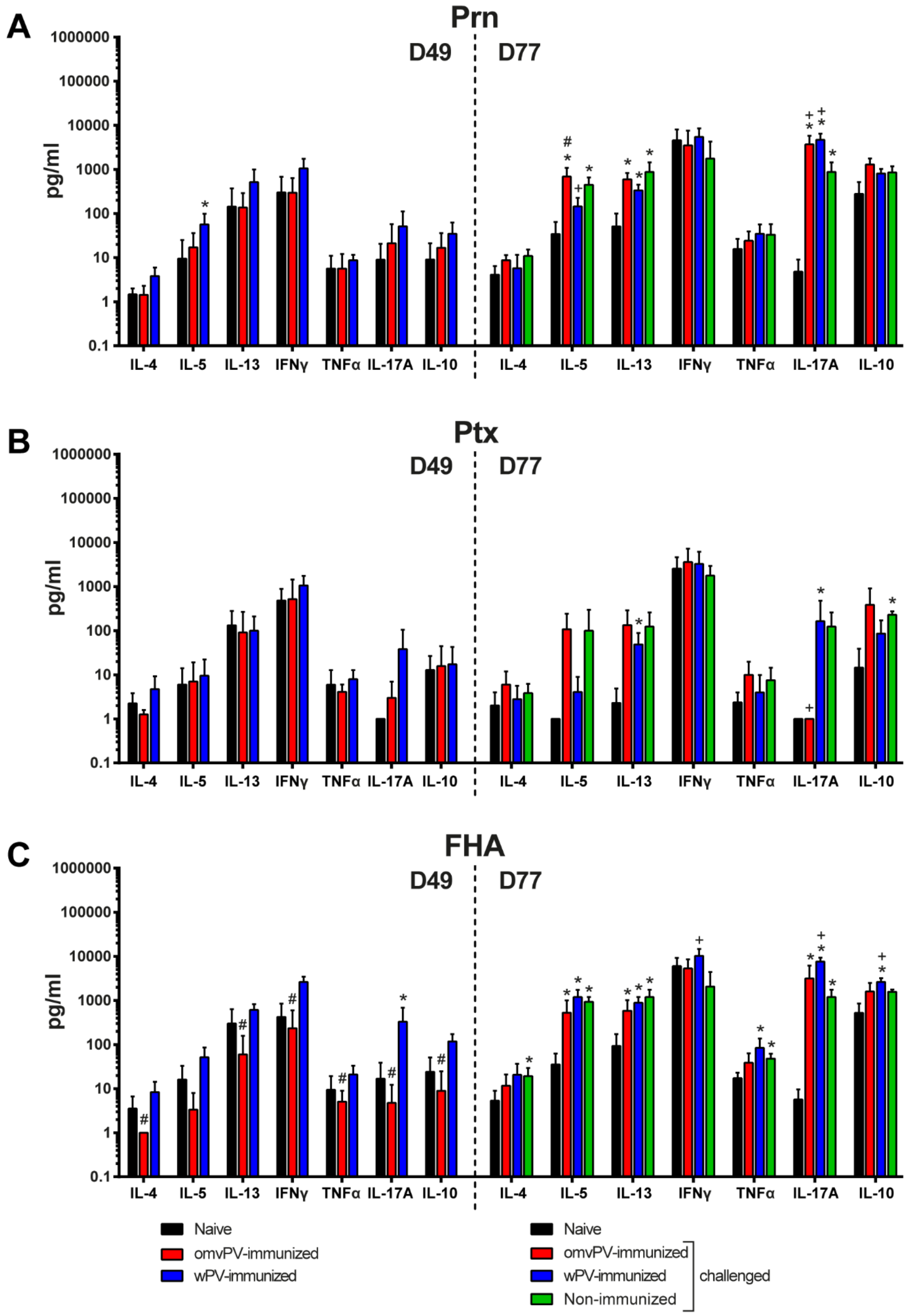

Supplementary Figure S1. Splenic cytokine responses after antigen restimulation in omvPV- and wPV-immunized mice as compared to naive control mice before and after challenge. (A-C) Concentrations of IL-4, IL-5, IL-13, IFNγ, TNFα, IL-17A and IL-10 were determined in the culture supernatants after 7 day stimulation of splenocytes with 1 µg/ml Prn, Ptx, or FHA. Splenocytes were harvested post booster immunization (day 49, left panel) of mice immunized with omvPV (red) or wPV (blue). Post-challenge (day 77, right panel), same groups were included with an additional group of non-immunized mice that received a challenge (green). In both experiments, complete naive mice (black) were used as control. Results for each mouse are corrected for medium stimulation. * = $p \leq 0.05$ for immunized group and challenged group vs. naive group, # = $p \leq 0.05$ for omvPV group vs. wPV group, + = $p \leq 0.05$ for challenged immunized group vs. challenge non-immunized group.

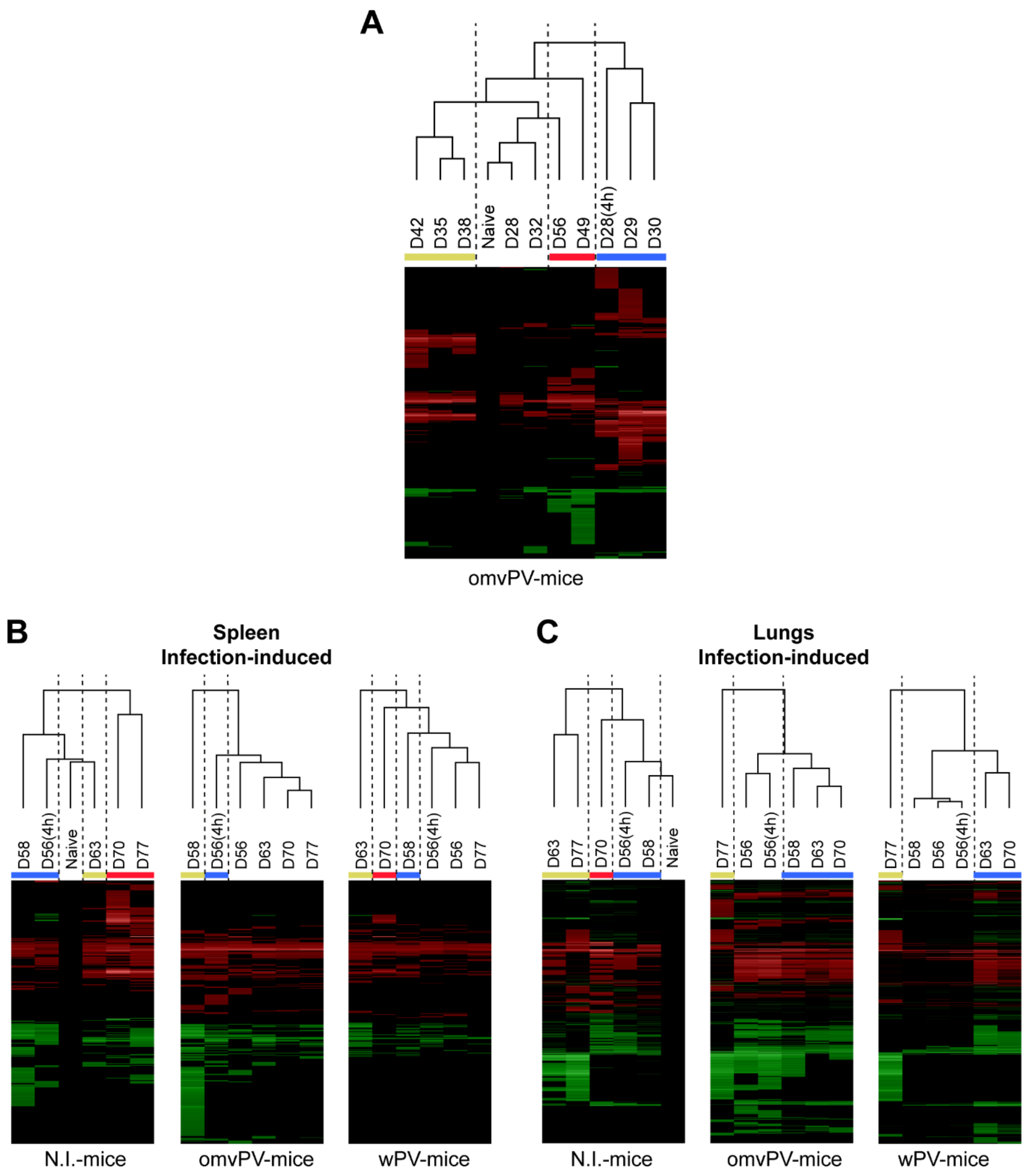

Supplementary Figure S2. Hierarchical clustering. (A) Hierarchical clustering of the omvPV-induced splenic transcriptome dataset to identify which time points showed a similar response. The response was divided in four parts illustrated by the different colors. (B-C) Hierarchical clustering on the (B) splenic and (C) pulmonary transcriptome datasets of challenged N.I.-mice, omvPV-mice and wPV-mice to identify which time points showed a similar response.

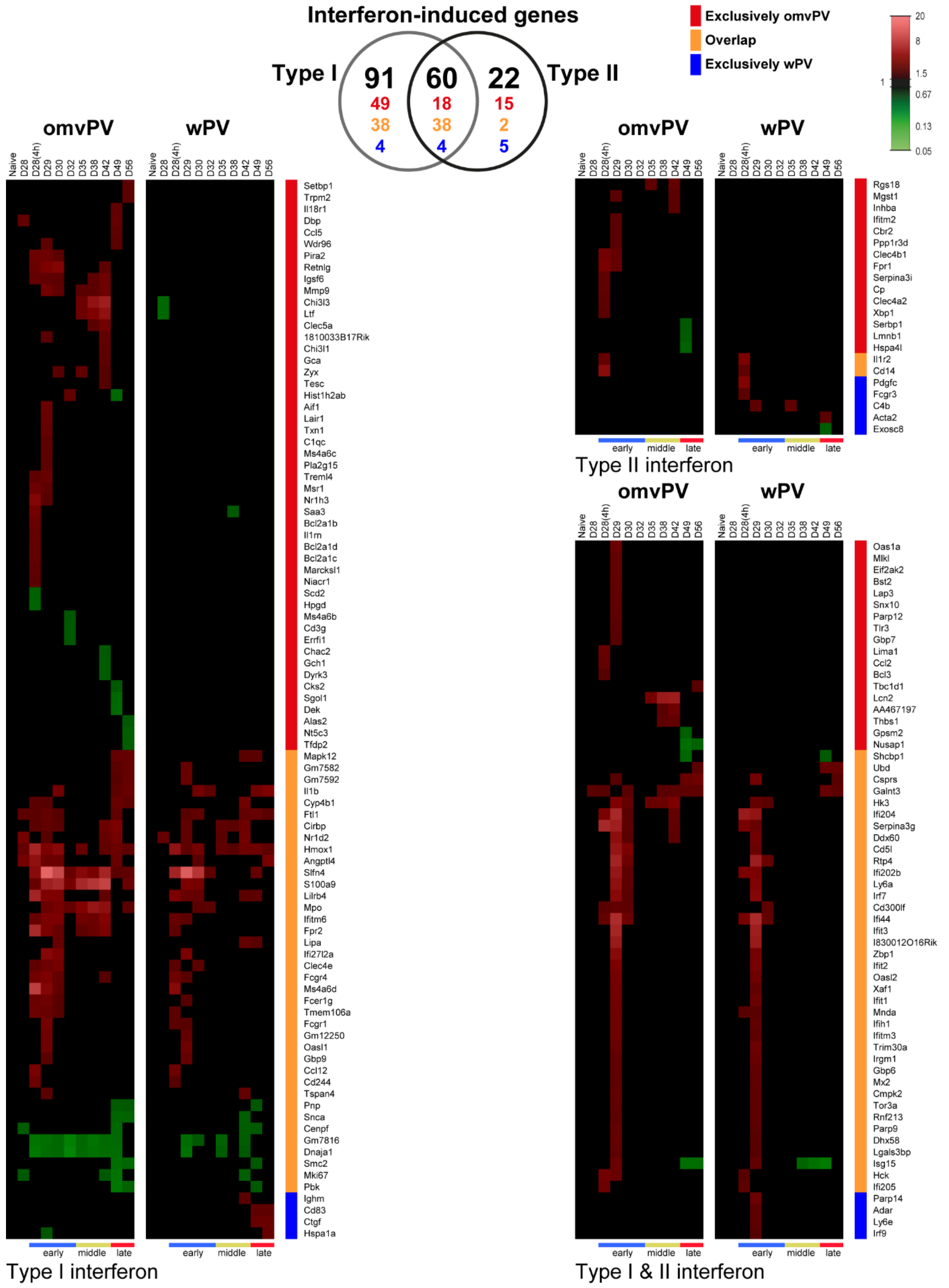

Supplementary Figure S3. IFN-induced genes in spleens of omvPV- and wPV-immunized mice. Genes induced by omvPV and wPV in the transcriptome of the spleen were matched with the Interferome database (http://www.interferome.org/interferome/home.jspx). A Venn diagram shows the total number of genes (black) induced by type I IFN, type II IFN, or both. Genes that were found exclusively in the omvPV-mice (red), wPV-mice (blue) or overlapped in both groups (orange) are depicted. The individual genes involved in type I IFN, type II IFN, or both are shown in heatmaps with the corresponding color codes of immunization background.

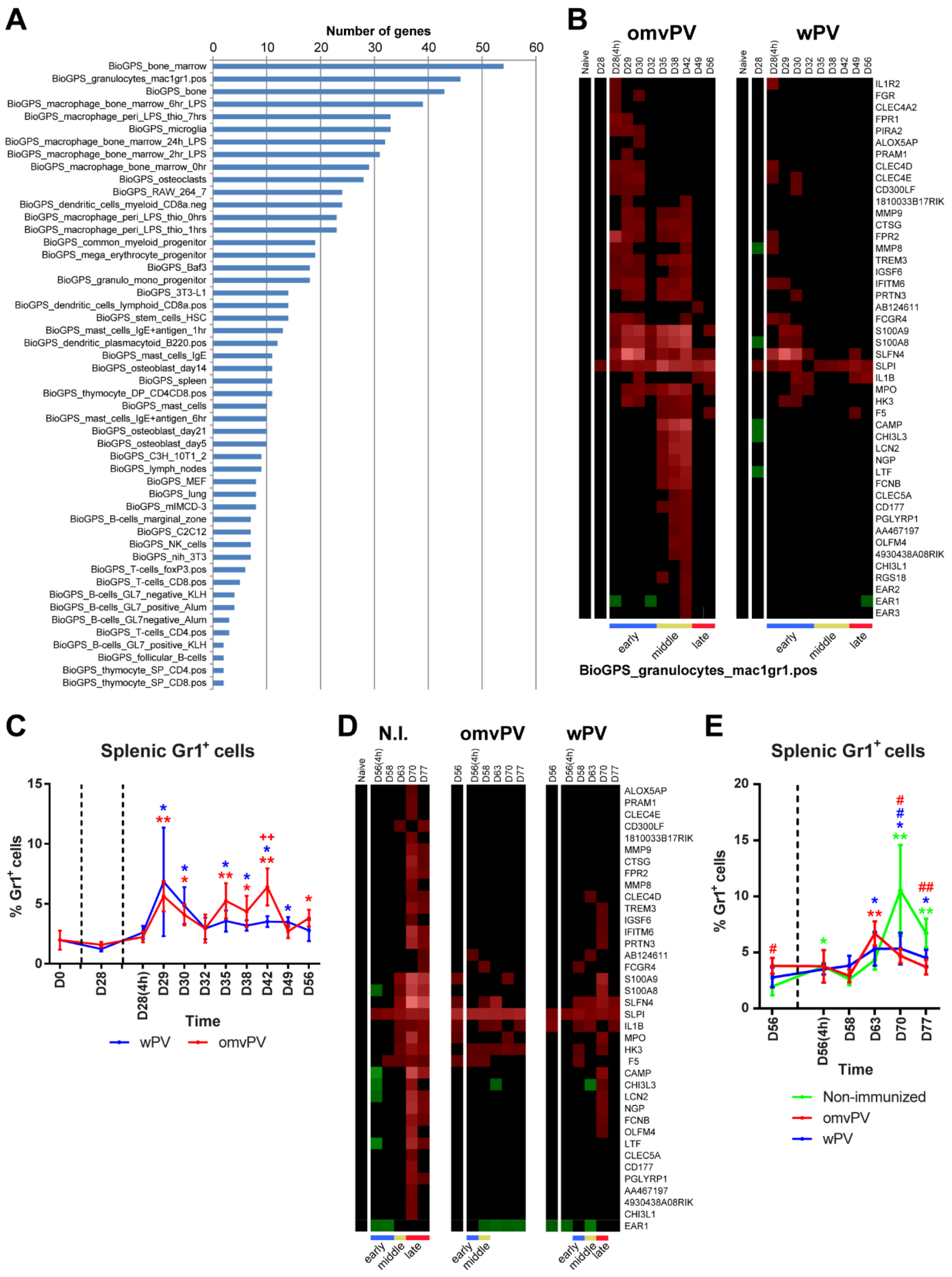

A
Number of genes
0 10 20 30 40 50 60
BioGPS_bone_marrow
BioGPS_granulocytes_mac1gr1.pos
BioGPS_bone
BioGPS_macrophage_bone_marrow_6hr_LPS
BioGPS_macrophage_peri_LPS_thio_7hrs
BioGPS_microglia
BioGPS_macrophage_bone_marrow_24h_LPS
BioGPS_macrophage_bone_marrow_2hr_LPS
BioGPS_macrophage_bone_marrow_0hr
BioGPS_osteoclasts
BioGPS_RAW_264_7
BioGPS_dendritic_cells_myeloid_CD8a.neg
BioGPS_macrophage_peri_LPS_thio_0hrs
BioGPS_macrophage_peri_LPS_thio_1hrs
BioGPS_common_myeloid_progenitor
BioGPS_mega_erythrocyte_progenitor
BioGPS_Baf3
BioGPS_granulo_mono_progenitor
BioGPS_3T3-L1
BioGPS_dendritic_cells_lymphoid_CD8a.pos
BioGPS_stem_cells_HSC
BioGPS_mast_cells_IgE+antigen_1hr
BioGPS_dendritic_plasmacytoid_B220.pos
BioGPS_mast_cells_IgE
BioGPS_osteoblast_day14
BioGPS_spleen
BioGPS_thymocyte_DP_CD4CD8.pos
BioGPS_mast_cells
BioGPS_mast_cells_IgE+antigen_6hr
BioGPS_osteoblast_day21
BioGPS_osteoblast_day5
BioGPS_C3H_10T1_2
BioGPS_lymph_nodes
BioGPS_MEF
BioGPS_lung
BioGPS_mIMCD-3
BioGPS_B-cells_marginal_zone
BioGPS_C2C12
BioGPS_NK_cells
BioGPS_nih_3T3
BioGPS_T-cells_foxP3.pos
BioGPS_T-cells_CD8.pos
BioGPS_B-cells_GL7_negative_KLH
BioGPS_B-cells_GL7_positive_Alum
BioGPS_B-cells_GL7negative_Alum
BioGPS_T-cells_CD4.pos
BioGPS_B-cells_GL7_positive_KLH
BioGPS_follicular_B-cells
BioGPS_thymocyte_SP_CD4.pos
BioGPS_thymocyte_SP_CD8.pos
B
omvPV
wPV
Naive D28 D28(4h) D29 D30 D32 D35 D38 D42 D49 D56
IL1R2
FGR
CLEC4A2
FPR1
PIRA2
ALOX5AP
PRAM1
CLEC4D
CLEC4E
CD300LF
1810033B17RIK
MMP9
CTSG
FPR2
MMP8
TREM3
IGSF6
IFITM6
PRTN3
AB124611
FCGR4
S100A9
S100A8
SLFN4
SLPI
IL1B
MPO
HK3
F5
CAMP
CHI3L3
LCN2
NGP
LTF
FCNB
CLEC5A
CD177
PGLYRP1
AA467197
OLFM4
4930438A08RIK
CHI3L1
RGS18
EAR2
EAR1
EAR3
early middle late
BioGPS_granulocytes_mac1gr1.pos
C
Splenic Gr1+ cells
% Gr1+ cells
0 5 10 15
D0 D28 D28(4h) D29 D30 D32 D35 D38 D42 D49 D56
Time
wPV omvPV
D
N.I.
omvPV
wPV
Naive D56(4h) D58 D63 D70 D77
D56 D56(4h) D58 D63 D70 D77
ALOX5AP
PRAM1
CLEC4E
CD300LF
1810033B17RIK
MMP9
CTSG
FPR2
MMP8
CLEC4D
TREM3
IGSF6
IFITM6
PRTN3
AB124611
FCGR4
S100A9
S100A8
SLFN4
SLPI
IL1B
MPO
HK3
F5
CAMP
CHI3L3
LCN2
NGP
FCNB
OLFM4
LTF
CLEC5A
CD177
PGLYRP1
AA467197
4930438A08RIK
CHI3L1
EAR1
early middle late
E
Splenic Gr1+ cells
% Gr1+ cells
0 5 10 15 20
D56 D56(4h) D58 D63 D70 D77
Time
Non-immunized
omvPV
wPV

Supplementary Figure S4. Involvement of Gr1$^+$ cells following omvPV and wPV booster immunization and after *B. pertussis* challenge. (A) Transcriptomic profiles from spleen tissue of omvPV-mice and wPV-mice were compared with BioGPS databases. The numbers of genes detected in the different BioGPS databases are listed. (B) Genes found following omvPV and/or wPV immunization that showed overlap with the Mac$^+$Gr1$^+$ granulocytes dataset. (C) Percentage of Gr1$^+$ cells in the spleen following omvPV and wPV immunization as determined by using flow cytometry. Data presented as mean ±SD (n = 4). * and ** = $p \leq 0.05$ and $p \leq 0.01$ for immunized mice vs. naive mice (day 0), ++ = $p \leq 0.01$ for omvPV-mice vs. wPV-mice. (D) Genes that matched with the Mac$^+$Gr1$^+$ granulocytes dataset in the transcriptomic profiles from spleen tissue of omvPV-, wPV-, and non-immunized mice following a *B. pertussis* challenge. (E) Percentage of Gr1$^+$ cells in the spleen after challenge of immunized and non-immunized mice immunization as determined by using flow cytometry. Data are presented as mean ± SD (n = 3). * and ** = $p \leq 0.05$ and $p \leq 0.01$ for challenged groups vs. day 56, # and ## = $p \leq 0.05$ and $p \leq 0.01$ for immunized groups vs. non-immunized group.

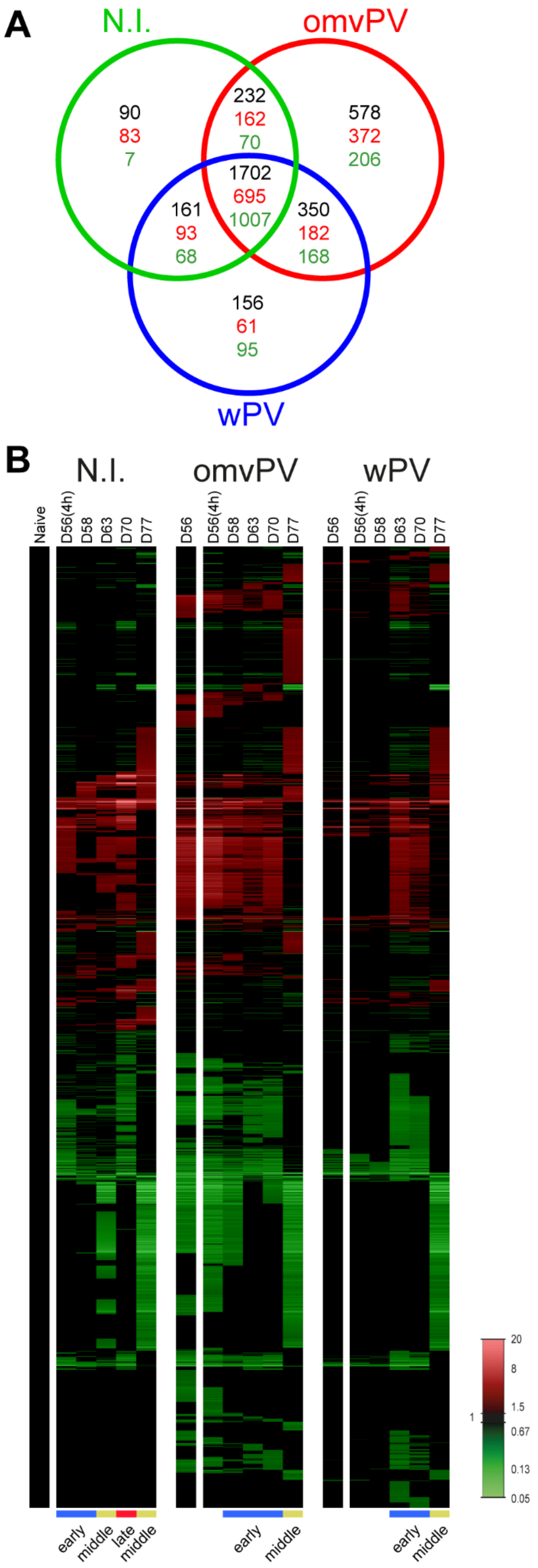

Supplementary Figure S5. Pulmonary transcriptomic profiles following *B. pertussis* challenge in omvPV-, wPV-, and non-immunized mice. (A) Fold changes in expression and significant gene expression were calculated compared to naive mice (FR ≥1.5, *p*-value ≤0.001). In total, 3269 DEGs were found divided over the three groups in a Venn-diagram with total number of genes (black), upregulated genes (red), and downregulated genes (green). (B) All differentially upregulated (red) and downregulated (green) genes are portrayed as heatmap (mean of n = 3 for immunized groups, n = 1 (pool of 3 mice for non-immunized group)). Genes not surpassing a FR of 1.5 are shown as basal level (black). Gene clustering is based on up/downregulation, time of involvement, and presence in the different groups. Infection-induced responses were divided in phases according to the hierarchical clustering calculated in Supplementary Fig. S2C.
